# Spontaneous left main coronary artery dissection complicated by pseudoaneurysm formation in pregnancy: role of CT coronary angiography

**DOI:** 10.1186/1749-8090-4-15

**Published:** 2009-04-01

**Authors:** Shahid Rahman, Mohammed Abdul-Waheed, Tarek Helmy, Lynn C Huffman, Vipin Koshal, Julian Guitron, Walter H Merrill, David F Lewis, Stephanie Dunlap, Yukitaka Shizukuda, Neal L Weintraub, Cristopher Meyer, Mehmet Cilingiroglu

**Affiliations:** 1 Mehmet Cilingiroglu, Department of Internal Medicine, Division of Cardiovascular Diseases, University of Cincinnati College of Medicine, 231 Albert Sabin Way, ML0542, Cincinnati, OH 45267-0542

## Abstract

We report a case of a 26-year-old female, who presented at 34 weeks of an uncomplicated pregnancy with an acute ST elevation anterior wall myocardial infarction. Cardiac catheterization suggested a left main coronary artery dissection with pseudoaneurysm formation. The patient's course was complicated by congestive heart failure. She was initially managed conservatively by a multidisciplinary team including heart failure specialists, obstetricians, and cardiovascular surgeons. 4 days after admission, her LMC was imaged by dual-source 64 slice Cardiac computed tomography, coronary dissection was identified extending to the lumen, and the presence of pseudoaneurysm was confirmed. She underwent subsequently a staged procedure, which included placement of an intra-aortic balloon pump, cesarean section, and coronary artery bypass grafting. This case illustrates the utility of coronary artery CT imaging to assess the complexity and stability of coronary artery dissections, thereby helping to determine the need for, and timing of revascularization procedures.

## Introduction

Spontaneous coronary artery dissection (SCAD) is a rare cause of acute myocardial ischemia [1]. It is defined as hemorrhagic separation of the coronary artery media with creation of a false lumen in the absence of precipitating factors. SCAD is often fatal. However, the prognosis is favorable for patients who survive the initial event [1-3]. Revascularization procedures and conservative medical management strategies have been applied in patients presenting with SCAD. Surgical intervention is indicated in cases of SCAD when there is propagation of the dissection plane or luminal narrowing resulting in myocardial ischemia with significant hemodynamic compromise [4].

Many published reports linked pregnancy with an increased risk for SCAD [2]. The underlying mechanisms are unclear but likely multifactorial in nature. In a recent review article, the mortality rate for pregnant patients presenting with SCAD was reported to be 38% [5]. There is no consensus opinion regarding whether to treat these patients medically or by revascularization. In this regard, the decision to perform diagnostic tests and revascularization procedures must also consider both maternal and fetal circumstances.

We present a clinical case of a pregnant patient presenting with SCAD of left main coronary artery (LMC) complicated by the formation of a pseudoaneurysm, which led to significant myocardial damage and onset of congestive heart failure. CT coronary angiography was used to document the persistence of dissection and pseudoaneurysm, thereby helping to guide the decision to proceed with surgical revascularization.

## Case presentation

**Figure 1:**
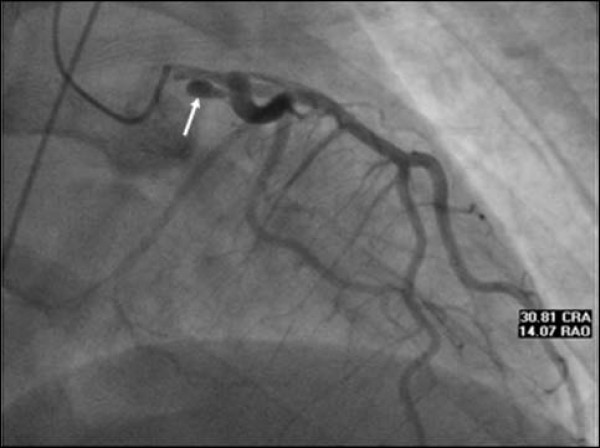
**Left coronary angiogram showing possible dissection of left main coronary artery with a pesudoaneurysm formation (White arrow)**.

A previously healthy 26-year-old female in the 34^th ^week of gestation was awakened from sleep by the sudden onset of severe substernal chest pain. On admission, blood pressure was 95/63 mmHg and, heart rate 87/min. An S4 detected on cardiopulmonary exam. EKG performed showed normal sinus rhythm with marked ST-elevations in leads V1–V4. She was taken to coronary angiography, which revealed LMC dissection with angiographic appearance suggesting a pseudoaneurysm, with moderate luminal compression of the distal vessel and TIMI-3 flow (Figure 1). Left ventriculography showed depressed ejection fraction (EF), a transthoracic echocardiogram confirmed akinesis of the apex, apical septum, mid-anteroseptum, and apical inferior walls with estimated EF approximately 30%. It was presumed that she had experienced a myocardial infarction secondary to embolic phenomenon from the pseudoaneurysm cavity. Chest pain had resolved fully, and she was started on intravenous heparin, aspirin, and low dose beta-blocker.

She was initially managed medically, as the infarction was felt to be complete and she was hemodynamically stable. Cardiac biomarkers were elevated and peaked soon after admission, with a creatine kinase of 5948 ug/L (normal range 0–165), creatine kinase-MB of 375 ug/L (normal range 0.2–5), and cardiac troponin I of 232 ug/L (normal range 0.01–0.05). While the presence of LMC dissection and pesudoaneurysm was concerning for recurrent embolic events, the risk to benefit ratio of performing urgent coronary artery bypass surgery (CABG) was difficult to estimate for several reasons; 1) she had suffered a large infarction, which would both increase the risk of urgent CABG and reduce the potential benefit of revascularization assuming that much of the at-risk myocardium was already infracted; 2) no residual thrombus was visible in the pseudoaneurysm cavity, and she was being treated with heparin and aspirin to prevent recurrent thrombus formation; 3) coronary dissections may spontaneously heal, without the need for revascularization.

She was managed with a multidisciplinary team including heart failure specialist, obstetricians, and cardiac surgeons. She developed clinical evidence of congestive heart failure, which responded to medical therapy, and fetal status remained stable under continous monitoring. She did not experience any recurrent chest pain.

**Figure 2:**
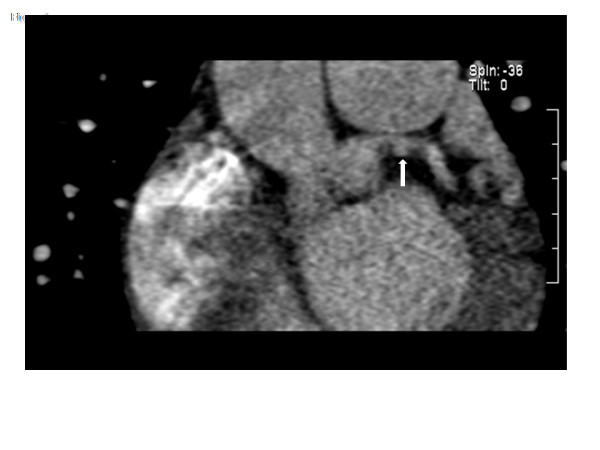
**Coronal oblique MPR images demonstrate the pseudoaneurysm and adjacent thrombus projecting inferior to the native left main coronary artery (White arrow)**.

**Figure 3:**
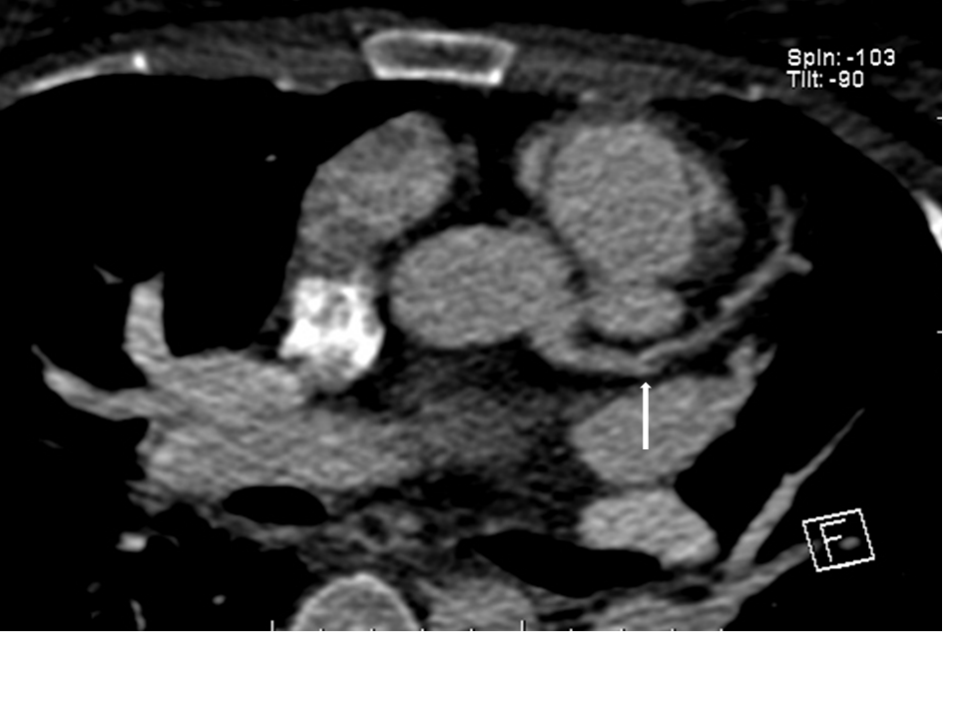
**Axial oblique images demonstrate the continuity of this pseudoaneurysm with an intimal flap identified in the proximal left anterior descending coronary artery (White arrow)**.

On day 4 post-infarction, high resolution imaging of LMC and left anterior descending coronary arteries was performed using a dual-source 64 slice CT scanner. The protocol was designed to limit radiation exposure, and she was placed on a continuous infusion of intravenous esmolol to slow heart rate. Cardiac CT showed LMC dissection flap extending into the coronary artery lumen and demonstrated the presence of a pseudoaneurysm (Figures 2, 3). Having shown the dissection did not heal spontaneously, she was prepared for a staged procedure with a cesarean section followed by CABG.

**Figure 4:**
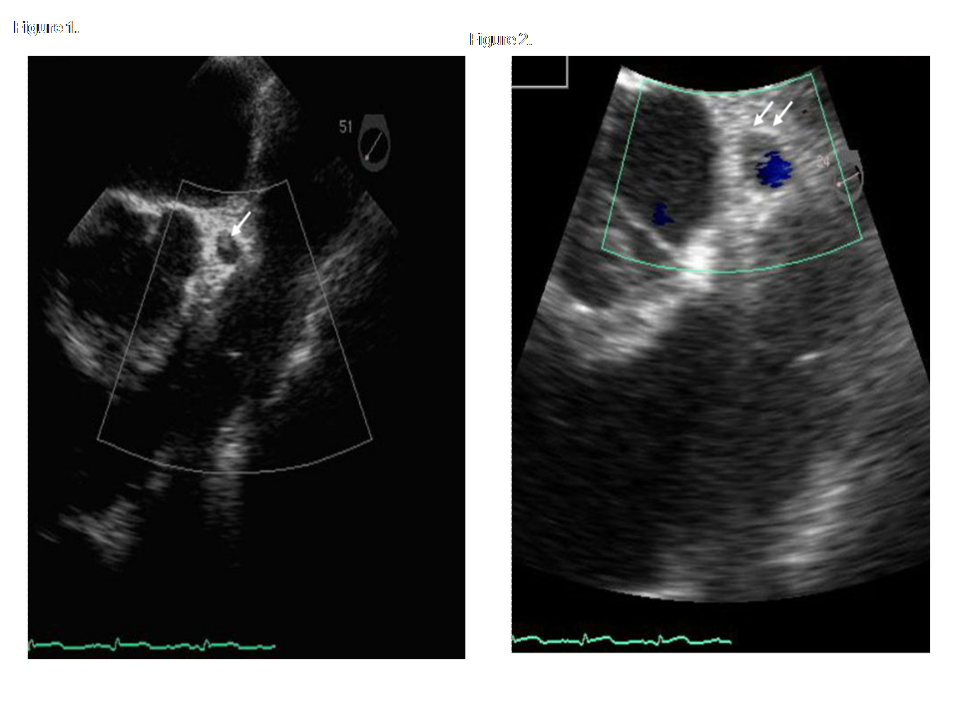
**The arrows indicate the echodensity noted in the left main coronary artery with transesophageal echocardiography**. Color Doppler flow Interrogation of the left main coronary artery indicate a significant stenosis due to this echodensity (left panel).

In preparation for surgery, a swan-ganz catheter was placed, during which she began complaining of substernal chest pain associated with dynamic ST-segment elevation on electrocardiogram. Intra-aortic balloon pump was inserted, and a transesophagial echocardiogram (TEE) was performed which showed an echodensity consistent with coronary artery thrombus occupying greater than 50% of the luminal area in LMC (Figure 4).

Subsequent to a successful low transverse cesarean section with bilateral tubal ligation, the patient underwent CABG. LMC was ligated, and the left anterior descending artery was grafted with the left internal mammary artery. The diagonal and obtuse marginal coronary arteries were revascularized using saphenous vein grafts. Postoperatively, she remained hemodynamically stable, and repeat echocardiogram performed 5 days post-operation showed no significant change in left ventricular function. At the time of delivery, the patient's cord blood was harvested, and stem cells were isolated and cryopreserved to use as a source of autologous cell-based therapy to improve cardiac function at a later date, if applicable.

At one month follow-up she had regained a great deal of her functional status, and was essentially asymptomatic on her medical regimen, which included carvedilol, lisinopril, digoxin, simvastatin, aspirin, warfarin, and furosemide.

## Discussion

We present a case of SCAD complicated by a pseudoaneurysm formation in a patient with no known cardiac risk factors who was 34 weeks pregnant. The exact pathophysiology of SCAD in this setting is unclear but it is thought to be related to hormonal changes associated with pregnancy. These hormonal changes may cause structural derangements in the vessel wall, including loss of elastic fibers, reticulin fiber fragmentation, hypertrophy of smooth muscle cells, and decrease in acid mucopolysaccharides [3,4]. Additional factors may contribute to the association between pregnancy and SCAD include increased shear forces associated with hyperdynamic state of gestation, and inflammation of the blood vessel wall [4]. Specific demographic trends have emerged in patients who are diagnosed with SCAD. More than 70% of the reported cases occurred in healthy young to middle aged women [6]. The peripartum state and use of oral contraceptives have been shown to be associated with increased incidence of SCAD [1,2].

Coronary pseudoaneurysms are quite rare and most frequently occur as a complication of percutaneous interventions [7]. Pseudoanuerysms may be difficult to distinguish from true aneurysms, which can be congenital in origin or acquired in the setting of atherosclerosis, vascular infections, vasculitis, cardiac tumors, or trauma. One case of coronary artery pseudoaneurysm consequent to SCAD in a pregnant patient was reported previously [8]. Given the limited information regarding pregnant patients presenting with coronary artery dissection complicated by pseudoaneurysm formation in the setting of myocardial infarction, no evidence-based data were available to guide decision making in this patient. As discussed previously, we initially elected to defer revascularization *in lieu *of medical therapy. However, once the decision was made to manage her conservatively, it was imperative to monitor her closely, with a multidisciplinary team of specialists, and to re-assess her coronary anatomy at a later time point to determine whether the lesion was persistent or had spontaneously healed.

Here we identified the novel use of cardiac CT in the evaluation of SCAD with pseudoanuerysm formation that obviated the need for repeat invasive coronary angiography. However, performing cardiac CT in a pregnant patient requires strict attention to the ALARA principals of radiation protection. It is critical to insure that not only the fetal dose of radiation is kept to a minimum but also the lactating breast dose is also minimized, since the breasts are most radiation sensitive during this time frame. For this reason, several methods of radiation protection were employed [9-11].

It is interesting to note that thrombus was detected by TEE in the pseudoaneurysm cavity at the time of surgery, despite the use of full-dose heparin and aspirin therapy. Whether this represented *de novo *thrombosis, or thrombus that was present from the outset but undetectable on prior imaging studies, can not be answered with certainty. We speculate that thrombus formation and/or persistence may have been facilitated by the hypercoagulable state of late-term pregnancy.

In pregnant patient with SCAD, the decision to pursue medical management, percutaneous coronary intervention, or surgical revascularization is based primarily on the clinical presentation, extent of dissection, and amount of ischemic myocardium at risk [4]. In the setting of a large myocardial infarction with congestive heart failure, an initial conservative strategy provides an opportunity to medically stabilize the patient, assemble a team of specialists to optimally care for both mother and baby, and plan the sequence of procedures. In such case, it is important to not only carefully observe the patient for signs of recurrent ischemia, but also to reassess the affected coronary artery. Our case illustrates the utility of cardiac CT as a non-invasive modality and identifies technical details that should be considered in balancing image quality with radiation protection for both mother and baby.

## Consent

A written informed consent was obtained from the patient for publication of this case report and accompanying images. A copy of the written consent form is available for review by the Editor-in-Chief of this journal.

## Competing interests

The authors declare that they have no competing interests.

## Authors' contributions

All authors contributed equally to the manuscript and all authors read and approved the final manuscript.
